# Molecular identification of *Trypanosoma theileri* complex in Eurasian moose *Alces alces* (L.)

**DOI:** 10.1016/j.ijppaw.2022.11.008

**Published:** 2022-11-21

**Authors:** Katarzyna Filip-Hutsch, Magdalena Świsłocka, Grzegorz Karbowiak, Anna W. Myczka, Aleksander W. Demiaszkiewicz, Joanna Werszko

**Affiliations:** a Department of Food Hygiene and Public Health Protection, Institute of Veterinary Medicine, University of Life Sciences (SGGW), Nowoursynowska 166, 02-787, Warsaw, Poland; b Department of Zoology and Genetics, Faculty of Biology, University of Białystok, Ciołkowskiego 1J, 15-245, Białystok, Poland; c Witold Stefański Institute of Parasitology, Polish Academy of Sciences, Twarda 51/55, 00-818, Warsaw, Poland

**Keywords:** *Trypanosoma* spp., Vector-borne pathogens, Epidemiology, Wild cervids

## Abstract

Although the significance of red deer (*Cervus elaphus*) and roe deer (*Capreolus capreolus*) as hosts and their role in the circulation of vector-borne pathogens in Europe is well described, the trypanosomes of moose (*Alces alces*) are poorly known. As heat sensitive ungulates, moose might be especially vulnerable to the effects of climate change and the associated rise in parasite pressure. Therefore, the aim of our study was to determine the prevalence of trypanosomes in moose in Poland, this being one of the largest populations in Central Europe since the 2001 hunting ban. Molecular analysis revealed the presence of *Megatrypanum* trypanosomes in almost half of the studied moose. As the population of moose in Central Europe has been recently growing, it is crucial to determine their role in the circulation of vector-borne pathogens in environment. This is the first study of the detection and molecular identification of *Trypanosoma theileri* complex in moose in central Europe.

## Highlights

• Morphological and molecular identification of *Trypanosoma* sp. in European moose.
• Two trypanosomes haplotypes belonging to the genus *Megatrypanum*.
• Similarity of sequences to trypanosomes isolated from cervids and tabanids.

## Graphical abstract

**Figure:**
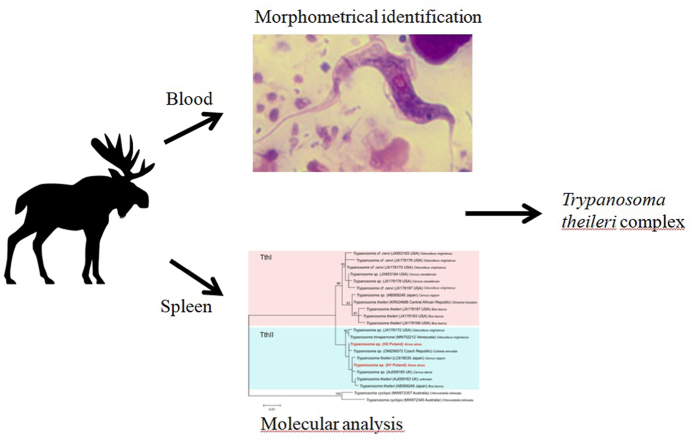

## 1 Introduction

Cervids are an zoonotically important group of mammals, serving as the animal reservoir for many pathogens known to cause the transmission diseases to humans and domestic animals (Karbowiak et al., 2015; Myczka et al., 2021). Unlike the well-described red deer and roe deer, the role of the moose remains undetermined.

The Eurasian moose (*Alces alces*) is the largest cervid species inhabiting Northern Europe, Asia and North America (Hundertmark and Bowyer, 2004). It has a Holarctic range, with its distribution covering the area from the Scandinavian Peninsula and the Baltic region, the northern part of Eastern Europe, Siberia and the Pacific Ocean, the northern part of Mongolia and China, as well as Alaska, Canada and the northern part of the USA. Presently, the western border of its European range, as well as the southernmost area of its distribution, runs through Poland (Schmölcke and Zachos, 2005; Świsłocka et al., 2020). Following the 2001 ban on moose hunting in Poland, their population has grown to 28 000 individuals; this now represents one of the largest groups of moose in central Europe, and the most crucial for their conservation (Wawrzyniak, 2016; Zalewski et al., 2018). As heat-sensitive ungulates, moose might be especially vulnerable to the effects of climate change (Borowik et al., 2020) and thus the growing pressure of vector-borne pathogens. Several studies indicate that moose act as hosts of *Babesia* piroplasms and *Anaplasma phagocytophilum* (Stuen et al., 2002; Karbowiak et al., 2015; Pūraitė et al., 2016; Myczka et al., 2021); they are also subject to infestation by bloodsucking arthropods able to transmit the pathogens, such as the tick *Dermacentor reticulatus* (Bogdaszewska, 2005; Karbowiak 2022) and the deer ked *Lipoptena cervi* (Paakkonen et al., 2010). However, very little is known about *Trypanosoma* spp. infection in moose (Kingston et al., 1985; Neumüller et al., 2012).

The genus *Trypanosoma* encompasses a number of parasitic protozoan species of human and animal health importance. Mammalian subgenera of trypanosomes are included in the terrestrial clade, together with avian subgenera, and subdivided into monophyletic Salivaria (subgenera: *Duttonella* Chalmers, 1918; *Nannomonas* Hoare, 1964; *Trypanozoon* Lühe, 1906; *Pycnomonas* Hoare, 1964) and polyphyletic Stercoraria (subgenera: *Megatrypanum* Hoare, 1964; *Herpetosoma* Doflein, 1901; *Schizotrypanum* Chagas, 1909; *Aneza* Özdikmen, 2009) (Kostygov et al., 2021).

Little is currently known of the trypanosomes of wild ruminants in temperature forest zones. They are classified to the subgenus *Megatrypanum* Hoare, 1972 (genus *Trypanosoma* Gruby, 1843), comprising a group of large trypanosomes able to infect almost all mammalian orders (Hoare, 1972). In central Europe, five species of the *Megatrypanum* subgenus have been described in ruminants based on morphological and morphometric data: *Trypanosoma wrublewskii* Wladimiroff and Yakimoff, 1909 in European bison *Bison bonasus* (L., 1758), *Trypanosoma theileri* Laveran 1902 in cattle *Bos taurus* L., 1758, *Trypanosoma stefanskii* Kingston et al., 1992a in roe deer *Capreolus capreolus* (L., 1758), *Trypanosoma cervi* Kingston and Morton, (1975) in red deer *Cervus elaphus* L., 1758, and *Trypanosoma melophagium* (Flu, 1908) in sheep *Ovis aries* L.,1758 (Kingston and Morton, 1975; Demiaszkiewicz and Lachowicz, 1991; Kingston et al., 1992a, b; Karbowiak et al., 2014).

The trypanosomes of European wild mammals have so far been detected using light microscopy, and their descriptions and characteristics are usually based on the morphological features visible on microscope slides. However, molecular tools and detailed phylogenetic analysis have provided a better understanding of the taxonomic relationships of *Megatrypanum* trypanosomes, revealing three main lineages of *T. theileri* (TthI, TthII and TthIII) and several genotypes (Rodrigues et al., 2006; Garcia et al., 2011, 2020; Brotánková et al., 2022). The purpose of this study was to confirm the presence of trypanosomes in moose in Poland and classify them based on a combination of molecular approaches and traditional methods.

## 2 Materials and methods

### 2.1 Study area

West Polesie (51°23′ N, 23°11′ E) is located in eastern Poland, on the border of Belarus and Ukraine, in the area of the Bug and Wieprz rivers. The region belong to temperature forest zone with typical continental climate, which features warm to hot summers and cold winters. The mean annual air temperature is 7.3 °C. Precipitation varies from 400 mm to 850 mm and most of the rain falls in the summer (Urban, 2005).

The Kampinos Forest (52°19′ N, 20°34′ E) is located in the Mazovian Lowland in central Poland and covers a part of the ancient valley of the Vistula basin. It lies in the temperate forest climate zone and is exposed to transitional marine and continental influences. The mean annual temperature is 7.7 °C and the precipitation is 547 mm (Andrzejewska, 2003).

### 2.2 Material collection

Post-mortem examinations were carried out on 13 moose, aged 1 month to 12 years, found dead in the wild or killed in road accidents in the years 2018–2021: ten in Kampinos Forest and three in West Polesie. The post-mortem examinations were performed in the field according to standard necropsy techniques and parasitological procedures, no more than two days after death. Age, sex and the body condition of the animals were determined. Spleen fragments were collected during the dissections, immediately transported to the laboratory and frozen at −20 °C until further analyses. In one case of a freshly-killed animal in the Kampinos Forest, it was possible to collect also a blood sample. The blood was collected in a heparin-coated tube, and stored at room temperature.

### 2.3 Microscopical study

In the blood sample, the trypanosomes were detected using the microhaematocrit centrifugation technique (8 min, 6200 g). The trypanosomes accumulated above the WBC fraction, and their movements were observed using light microscopy, at magnifications 10× 10 and 10 × 20 (eyepiece × objective). Smears were performed from the trypanosome fraction. The smears were fixed in methanol (10 min). As the smears performed from the centrifuged blood did not stain well with Giemsa's reagent, the commercial Hemacolor® kit (Merck, Germany) was used. Parasite measurements were performed using the Olympus BX50 light microscope. Stained blood smears were analyzed at a magnification of 1200 × ,using the Cell D digital image analysis software (Olympus Europe). The nomenclature of the morphometric parameters of trypanosomes was that commonly used by other authors (Hoare, 1972; Matthews et al., 1977; Kingston et al., 1992a, b).

### 2.4 Molecular analysis

DNA from spleen samples, about 20 mg weight, was extracted using the AX Tissue Mini kit (A&A Biotechnology, Gdynia, Poland) according to the manufacturer's protocol and stored at −20 °C until further laboratory analysis. To molecular detect the trypanosomes in the spleen samples, nested-PCR with primers (TRY927F, TRY927R and SSU561F, SSU561R) according to Noyes et al., (1999) were used to amplify *18S* rRNA partial gene.

A 4 μl DNA template was used for the primary reactions and 1 μl of amplification product for the nested amplifications. For both reactions, Taq DNA Polymerase (EURx, Gdańsk, Poland) was used and the amplification was performed according to Noyes et al. (1999). The presence of 523 bp reaction products were considered positive. DNA from cultured *Trypanosoma* sp. (GenBank accession number: KJ397590) isolated from the blood of red deer was used as a positive control, while the negative control consisted of nuclease-free water added to the PCR mix instead of the DNA sample.

Nested PCR products were visualized on 1% agarose gels stained with SimplySafe™ (EURx, Gdańsk, Poland). The gels were visualized using ChemiDoc, MP Lab software (Imagine, BioRad, Hercules, USA). The positive products of nested PCR were purified using the Agarose-Out DNA Purification Kit (EURx, Gdańsk, Poland), and sequenced by Genomed (Warsaw, Poland). The obtained sequences were assembled using ContigExpress, Vector NTI Advance 11.0 (Invitrogen Life Technologies, New York, USA), aligned with reference sequences available in GenBank by BLAST (NCBI, USA) and analyzed.

The *Trypanosoma* sp. *18S* rRNA gene sequencing results were aligned and revised manually using BioEdit v7.0.4 (Hall, 1999). Any *18S* rRNA gene sequences obtained in our study were submitted to GenBank. To test the phylogenetic relationships among our newly-obtained *18S* rRNA haplotypes of *Trypanosoma* sp. and sequences downloaded from GenBank, a phylogenetic tree was constructed using a maximum likelihood (ML) algorithm in Mega v6.06 (Tamura et al., 2013) using 1000 bootstrap replicates. The GTR + I + G model of substitution was selected as the best-fitting model by the AIC test (Akaike Information Criterion) with the jModelTest (Posada, 2008) for the ML tree. The tree was rooted using sequences of *T. cyclops* downloaded from GenBank (GenBank accession no. MW872345 and MW872357; Ellis et al., 2021).

## 3 Results

### 3.1 Morphological identification

**Fig. 1:**
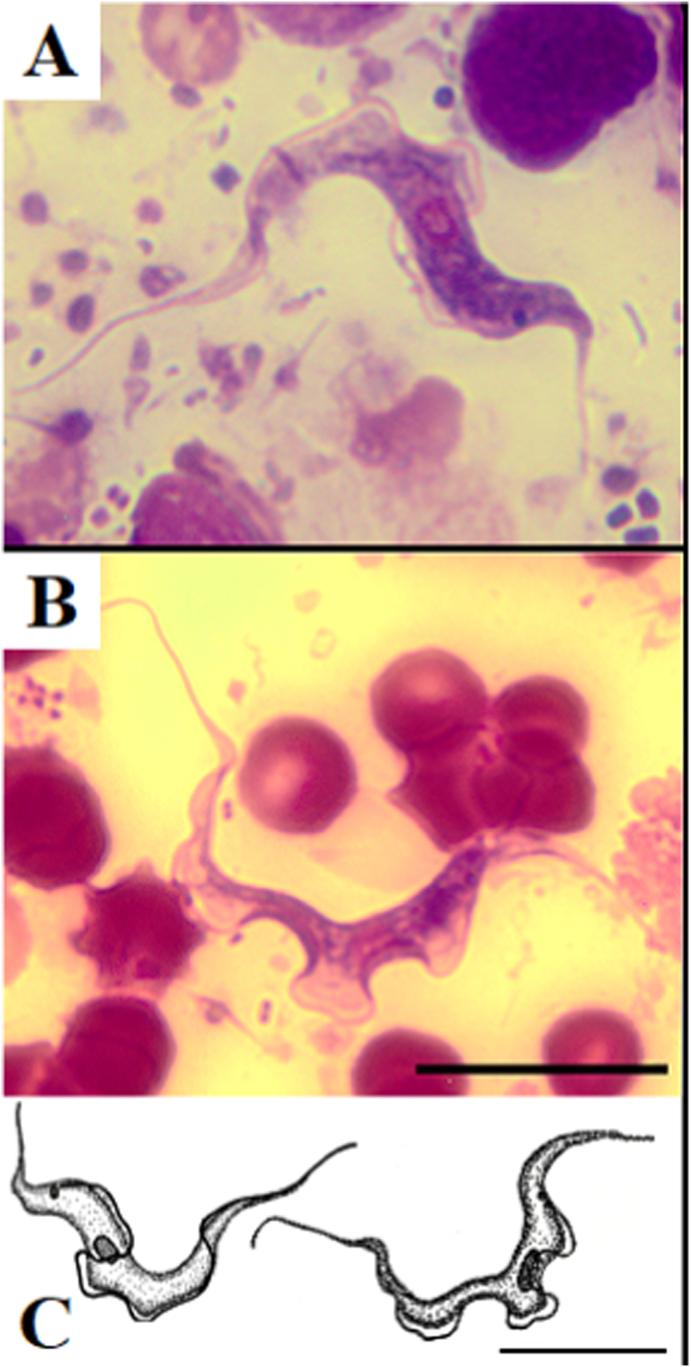
The trypanosomes from European moose. A, B. light microscope images; C. drawing scheme. Scale bar 10 μm.

Microscope examination confirmed the presence of three trypanosomes in the blood sample of an animal from Kampinos Forest (Fig. 1). As the analysis was performed in 750 μl of blood, the infection level can be estimated as four individuals per 1 ml. Morphological characterization show trypomastigote forms, typical for *Megatrypanum* (Fig. 1). All measurements are included in Table 1. The detected form had a body length (BL) of 33.8–37.4 μm, and body width (W) of 2.7–2.9 μm. Both ends of the body are elongated and sharpened. The free flagellum (FF) is relatively long, 6.5–9.8 μm in length, index FF/BL = 3.8–5,2. The undulating membrane is wide. The ovate nucleus (N) is 2.2–2.9 μm in size, located near the centre of the body (index NI = 0.9 to 1.1; posterior end to nucleus centre (PN) – 16.5 to 18.9; nucleus centre to anterior end (NA) – 17.3 to 18.6), kinetoplast located slightly closer to the nucleus than to the end of the cell (index KI = 2.6 to 3.1; posterior end to kinetoplast (PK) – 10.4 to 11.7; kinetoplast to nucleus centre (KN) – 6.1 to 7.1). The cytoplasm has no visible granules. Due to the low number of specimens, statistical analysis was not possible.

**Table 1:** Mean measurements (in μm) and size indices of *Trypanosoma* sp. from stained films of the moose, obtained in Kampinos Forest (Poland). Measurements: PK - posterior end to kinetoplast, KN - kinetoplast to nucleus centre, PN - posterior end to nucleus centre, NA - nucleus centre to anterior end, BL - body length, FF - free flagellum length, L - total length, N - nucleus length, W - width of body on the nucleus level excluding the undulating membrane. Indices: nuclear index NI = PN/NA, kinetoplastic index KI = PN/KN, flagellar index FF:BL.

	Parameter						
		PK	KN	PN	NA	BL	FF
*Trypanosoma* sp*.*	mean	11.06 ± 0.89	6.63 ± 0.72	18.09 ± 1.33	17.97 ± 0.90	35.07 ± 2.05	8.14 ± 2.28
*n* = 3	range	10.43–11.68	6.12–7.14	16.55–18.89	17.33–18.61	33.88–37.44	6.53–9.76

	Parameter						
		L	N	W	NI	KI	FF:BL
*Trypanosoma* sp.	mean	44.61 ± 3.67	2.55 ± 0.50	2.77 ± 0.14	1.02 ± 0.07	2.81 ± 0.24	4.74 ± 0.78
n = 3	range	40.41–47.19	2.20–2.90	2.67–2.86	0.95–1.09	2.64–3.09	3.84–5.19

### 3.2 Molecular analysis

Trypanosome DNA was detected in six out of thirteen investigated moose spleens: two from West Polesie and four from Kampinos Forest. They were recovered from three infected males and three infected females aged 3–12 years, including moose specimen, which blood was investigated using microscopic method described above. All uninfected individuals were under 6 months old.

The molecular analysis of the *18S* ribosomal RNA partial gene (523 bp) amplified from six studied *Trypanosoma* sp. yielded two haplotypes (GenBank accession numbers for haplotype 1 - ON870924, and haplotype 2: ON870925). Four sequences of *Trypanosoma* sp. from moose in Kampinos Forest were found to be identical the sequence from an animal in West Polesie (haplotype H1), whereas sequence isolated from another moose in West Polesie differed by one polymorphic site (haplotype H2).

BLAST sequence analysis of the obtained sequences found haplotype H1 to share 100% identity with *Trypanosoma* sp. from the horsefly *Hybomitra tarandina* in Russia (GenBank accession no. MK156791, Ganyukova et al., 2018; OL855998, OL856000, Kostygov et al., 2022) and 99.81% similarity with haplotypes found in *Simulium* sp. in the Czech Republic (GenBank accession no: OM256575, OM256576, Brotánková et al., 2022).

**Fig. 2:**
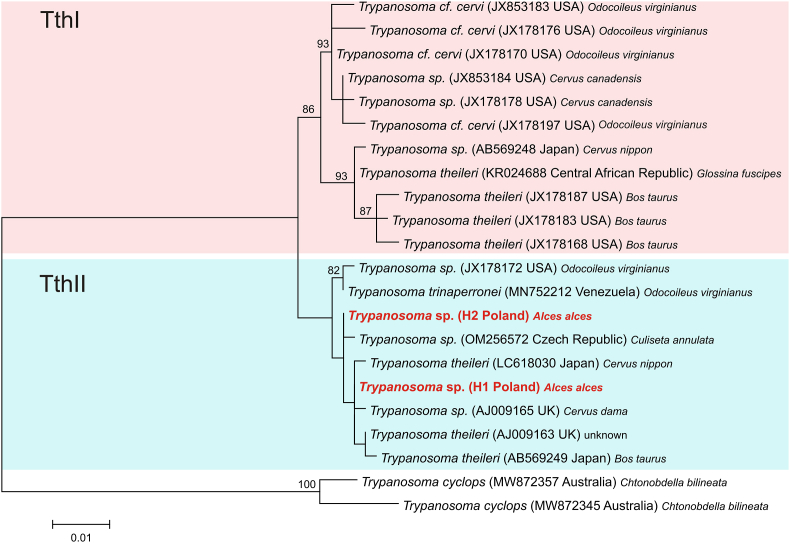
Phylogenetic tree of *Trypanosoma* sp. *18S* rRNA partial gene. Maximum-likelihood tree computed with the GTR + I + G model of sequence evolution. *Trypanosoma* sp. found in our study (haplotype H1 and H2 marked with red color) and downloaded from GenBank. Hosts were listed after GenBank numbers and country of origin. Numbers listed at nodes represent percent support for that node from 1000 bootstrap replicates. The ML tree has been rooted with sequences of *Trypanosoma cyclops*. (For interpretation of the references to color in this figure legend, the reader is referred to the Web version of this article.)

The maximum likelihood (ML) phylogenetic reconstructions produced a strong topology (Fig. 2), indicating that our two identified haplotypes belong to the group TthII. The *18S* rRNA haplotype H1 was grouped with haplotypes described for *Trypanosoma theileri* and *Trypanosoma* sp. from cattle and fallow deer in the United Kingdom (GenBank accession no. AJ009163, AJ009165, Stevens et al., 1998), and *T*. *theileri* isolated from sika deer in Japan (GenBank accession no. LC618030, Rosyadi et al., 2021). Haplotype H2 was grouped with *Trypanosoma* sp. isolated from the mosquito *Culiseta annulata* in the Czech Republic (GenBank accession no: OM256572, Brotánková et al., 2022).

## 4 Discussion

Trypanosome DNA was detected in the spleens of almost half the examined moose, including the animal in which trypanosomes have been additionally found in the blood by microscopic methods. This may indicate a relatively high protozoan prevalence in the studied moose population. Although further study based on more animals is needed to draw a fuller conclusion, the combination of high prevalence and low level of infection is typical for the trypanosome group (Kingston et al., 1992a, b; Wita and Kingston 1999). All uninfected individuals were under 6 months old, which might be a result of different immunological status of young moose or limited exposure to the parasite, as it was observed in relation to other trypanosomes (Whitelaw and Urquhart, 1985; Garcia et al., 2002).

As no statistical analysis was possible, it is difficult to compare the obtained metric data with the existing characteristics of *Megatrypanum* trypanosomes. Existing data on moose trypanosomes is sparse and tends to be very general. Wita et al. (2001) identified their studied moose parasites as *T. cervi* Kingston and Morton, (1975) (L = 44.18, NI = 0.89, KI = 2.12, FF:BL = 5.24). The trypanosomes found in this study are within the size range reported for *T. cervi* from red deer from Poland (Wita and Kingston, 1999). Based on present morphological analysis, and the fact that the specimens were obtained from moose, it is possible that our trypanosomes were examples of *T. cervi*; however this identification requires further confirmation and more detailed statistical analysis.

According to recent data a wide range of *Megatrypanum* trypanosomes should be considered as a *T. theileri* species complex consisting of three lineages, *viz.* TthI, TthII and TthIII, for parasites isolated from bovids and cervids (Rodrigues et al., 2006; Garcia et al., 2011; Martinković et al., 2012; Garcia et al., 2020; Brotánková et al., 2022). Phylogenetic analysis of the partial *18S* rRNA gene found haplotypes from this study H1 and H2 placed into the lineage TthII, together with isolates from Europe and Asia. Haplotype H1 was included in the subclade together with trypanosomes isolated from domestic cattle and wild cervids, belonging to the subgenus *Megatrypanum*. This protozoan group is considered typical for wild and domestic ruminants in Europe (Werszko et al., 2020 b, Werszko et al., 2020a), which was also confirmed in our study. *Megatrypanum* trypanosomes have been detected in cattle and wild bovids in Belgium, Italy, Ireland and Spain (Doherty et al., 1993; Verloo et al., 2000; Villa et al., 2008; Amato et al., 2019; Bittner et al., 2019) as well as in wild cervid species, including roe deer, fallow deer and red deer in Sweden, Germany and Austria (Friedhoff et al., 1984; Hoffmann et al., 1984; Hinaidy, 1987; Garcia et al., 2011; Neumüller et al., 2012); however, little data exists about their occurrence in Eurasian moose (Kingston et al., 1985; Neumüller et al., 2012). *Megatrypanum* trypanosomes are generally non-pathogenic, causing only subclinical infection (Matsumoto et al., 2011; Magri et al., 2021), which might be one of the reasons for random detection of the parasite in some cervid species.

Phylogenetic analysis revealed that haplotype H2 was grouped together with *Trypanosoma* sp. from the mosquito *Culiseta annulata* in the Czech Republic (Brotánková et al., 2022). *Trypanosoma theileri*-like trypanosomes were documented in the mosquitos previously (Schoener et al., 2018); however their role in the parasite transmission was not evidenced.

Morphometrical studies of various authors show the difference between trypanosomes parasitizing bovids and cervids, classifying them to the species *T. theileri* and *T. cervi*, respectively (Hoare 1972; Friedhoff et al., 1984; Böse et al., 1993). The distinctness of these lines has been recently confirmed by molecular studies in hosts inhabiting the American continent (Fisher et al., 2013; Kostygov et al., 2021). However in Europe limited molecular data on trypanosomes as well as possibility of one ruminant species being a host for different genetic lines of the parasite (Garcia et al., 2020; Rosyadi et al., 2021) hamper the interpretation of the results and unambiguous classification of the obtained sequences. It is possible that trypanosomes of moose in the present study might be considered as *Trypanosoma* cf. *cervi* or *T. theileri*; however it requires further study, including isolates from the entire range of European cervids and bovines.

Our findings expand previous knowledge about protozoans in cervids and provide a more extensive molecular and morphological characterization of moose trypanosomes. Further studies of the *Trypanosoma theileri* complex are necessary to determine the spread of the parasite in the population of wild cervids in Europe.

## Funding

This research did not receive any specific grant from funding agencies in the public, commercial, or not-for-profit sectors.

## Declaration of competing interest

The authors declare that they have no known competing financial interests or personal relationships that could have appeared to influence the work reported in this paper.
